# Correction: Shahid et al. Proximate Composition and Nutritional Values of Selected Wild Plants of the United Arab Emirates. *Molecules* 2023, *28*, 1504

**DOI:** 10.3390/molecules31071150

**Published:** 2026-03-31

**Authors:** Mohammad Shahid, Rakesh Kumar Singh, Sumitha Thushar

**Affiliations:** Division of Crop Diversification, International Center for Biosaline Agriculture, Dubai P.O. Box 14660, United Arab Emirates

**Citation Correction**

In the original publication [1], there was an error in the citation. Reference 1 in Introduction (Section 1) was previously as follows:

Madouh, T.A.; Al-Sabbagh, T.A. Nutritional quality and adaptation of several native plant species of Kuwait to the farming system as potential livestock feed. *Int. J. Env. Earth Sci*. **2021**, *2*, 1–8.

This is now corrected to:

Madouh, T.A. Eco-Physiological Responses of Native Desert Plant Species to Drought and Nutritional Levels: Case of Kuwait. *Front. Environ. Sci.* **2022**, *10*, 785517. https://doi.org/10.3389/fenvs.2022.785517.

**Text Correction**

The corresponding sentences in Introduction (Section 1) has been corrected as follows:

The desert ecosystems in the Arabian region impose multiple abiotic stresses on vegetation, including chronic water scarcity, high temperatures and solar radiation, soil salinity, and low nutrient availability, which together limit plant growth and establishment. However, native desert flora has evolved physiological and morphological adaptations that enable persistence under these harsh conditions [1].

The authors state that the scientific conclusions are unaffected. This correction was approved by the Academic Editor. The original publication has also been updated.
