# Supplementary material for: Proximate Composition and Nutritional Values of Selected Wild Plants of the United Arab Emirates
Source: Molecules. 2023 Feb 3;28(3):1504. doi: 10.3390/molecules28031504 (PMC9921872; doi:10.3390/molecules28031504)
Supplement: Supplementary file 1 [file molecules-28-01504-s001.zip › molecules-2100205-supplementary.pdf]

molecules
MDPI

Supplementary material

Detailed methodologies for the analysis of the chemical composition of selected wild plants of the United Arab Emirates

The chemical and nutritional components of seven wild plant species from UAE were analyzed by proximate composition, micronutrients, amino acids, and fatty acids content. The aerial parts of the samples were collected from ICBA campus, and the cleaned samples were transported to the Food Allergens Laboratory, Cyprus (ISO/IEC 17025:2017) (https://www.foodallergenslab.com/#!/en) in zip lock bags for analysis. The quantity per sample was 1 kg. The samples are processed mechanically. The detailed protocols for the analysis of the chemical composition are provided below

- Fat content (Fat) was determined by the Soxhlet extraction method as described by [81].
- Protein (PS) was measured by the Kjeldahl method, as described by [81].
- Moisture content was determined by the method described by [81].
- Ash content (Ash) was analyzed after burning the plants in a muffle furnace [81] .
- Crude fiber (CF) content was determined using the neutral detergent reagent method [82].
- Total carbohydrate (CHO) content was estimated by the difference between 100 and the sum of the percentages of moisture, protein, total lipid, and ash contents [83].
- Minerals content: potassium (K), calcium (Ca), phosphorus (P), and zinc (Zn), were analyzed using the Inductive Coupled Plasma (ICP) Emission spectrometer according to [83].
- Vitamins content: Vitamins, A C, B1, B3 and B6 were measured based on high-performance liquid chromatography (HPLC) analysis as follows:
    - a) Device Specification: Waters 2690 Alliance HPLC system equipped with a Waters 996 photodiode array detector.
    - b) Standard preparation: Weigh 10mg of 7 water soluble vitamins reference standards (Ascorbic acid, Thiamine HCl, Riboflavin, Nicotinic acid, Nicotinamide, Pyridoxine HCl and Folic acid) in 10ml 0.05 M NaOH, dilute to concentration 100µg/ml and solution was filtered on 0.22 µm syringe filter then 100 µl was injected.
    - c) Sample preparation: Five Extracts were prepared with concentration of 50mg/ml, filtered on 0.22 µm syringe filter then 100 µl was injected.
    - d) HPLC analysis conditions: • Column Inertsil ODS 3: 4.6x250mm, 5µm •Mobile phase: (0.85gm Hexane sulphonic acid in 1000 ml water and adjust pH to 3 with orthophosphoric acid): Methanol • Mode of elution: Gradient • Flow rate: 1 ml/min • Temperature: Ambient • Wavelength: 230 nm
- Amino acids were determined using Sykam Amino Acid Analyzer (Sykam GmbH, Germany) as follows:
    - a) Device Specification: Sykam Amino Acid Analyzer (Sykam GmbH, Germany) equipped with Solvent Delivery System S 2100 (Quaternary pump with flow range 0.01 to 10.00 ml/min and maximum pressure up to 400 bar), Autosampler S 5200, Amino Acid Reaction Module S4300 (with built-in dual filter photometer between 440 and 570 nm with constant signal output and signal summary option) and Refrigerated Reagent Organizer S 4130.
    - b) Standard preparation: Stock solution contains 18 Amino acids (Aspartic acid, Threonine, Serine, Glutamic acid, Proline, Glycine, Alanine, Cysteine, Valine, Methionine, Isoleucine,

Leucine, Tyrosine, Phenylalanine, Histidine, Lysine, Ammonia, Arginine) all amino acids concentrations are 2.5µMol/ml, except Cystine 1.25µMol/ml, then dilute 60 µl in 1.5 ml vial with sample dilution buffer then filtered using 0.22 µm syringe filter then 100 µl was injected.

  - c) Sample preparation: 1 gm of each sample was mixed with 5 mL hexane. The mixture was allowed to macerate for 24 hours. Then, the mixture was filtered on whatman no. 1 filter paper and the residue was transferred into a test tube where it was incubated in an oven with 10 mL 6N HCl for 24h at 110°C. After the incubation, the sample was filtered on whatman no. 1 filter paper, evaporated on rotary evaporator and dissolved completely in 2 ml dilution buffer. From this solution, the first dilution was prepared by diluting 100 µL to 1 mL dilution buffer and from which 10µL were further diluted to 1mL dilution buffer, filtered using 0.22 µm syringe filter and 100 µl was injected.
  - d) Instrument parameters: • Column: LCA K06/Na • Mobile phase: Buffer A, Buffer B and Regeneration solution • Mode of elution: Gradient •Flow rate: 0.45 ml/min • Temperature: Gradient 57ºC - 74ºC • Wavelength: 440 and 570 nm

- Fatty acid composition: The fat extracted from the samples was further analyzed for the fatty acids composition using the gas chromatography method. Identification of each fatty acid was conducted using the equivalent chain lengths and laboratory standards. Preparation of fatty acid methyl ester (FAME) was carried out according to [82]. A standard mixture of fatty acid methyl esters was used to identify of the peaks by their retention time. The different fatty acid methyl esters (FAMEs) were determined and identified using a gas chromatography (Hewlett Packard 6890) equipped with a flame ionization detector (FID). A HP-5 column (30m, 0.32mm ID, 0.25µm film thickness) [5% diphenyl, 95% dimethyl polysiloxane] was used. The detector and injector temperatures were 280ºC and 220ºC, respectively. Sample size 3 µl, spilt ratio 50:1. Nitrogen was used as carrier gas at a flow rate of 1 ml/min. Oven temperature was programmed as: - Set point (initial temperature) 150ºC for 2 Min. - Rate 10ºC/min to 200ºC. - Rate 5ºC/min to 250°C and hold for 9 Min. The concentrations of fatty acids were calculated as following equation: Fatty acid% = peak area ÷ overall area of peaks × 100.
